# Effect of Fiberglass Post Technique Removal on the Root Dentin Loss, the Root Canal Alignment and the Root Perforation Occurrence

**DOI:** 10.1590/0103-644020235851

**Published:** 2024-12-06

**Authors:** Marcio Alex Barros Gomes, Camilla Christian Gomes Moura, Lucas Raineri Capeletti, Gustavo Silva Chaves, Daniel de Almeida Decurcio, Monise de Paula Rodrigues, Carlos José Soares

**Affiliations:** 1 Department of Operative Dentistry and Dental Materials, Dental School, Federal University of Uberlândia, Uberlândia, Minas Gerais, Brazil; 2 Department of Endodontics, Dental School, Federal University of Uberlândia, Uberlândia (UFU), Minas Gerais, Brazil; 3 Aria Institute Brasília (ARIA), Brasília, Distrito Federal, Brazil; 4 Department of Endodontics, Dental School, Federal University of Goiás (UFG), Goiânia, Goiás, Brazil

**Keywords:** Adhesives, Argon plasma, Microtensile bond strength, Dentin, Scanning electron microscopy

## Abstract

Root canal retreatment and fracture of fiberglass posts (FGP) can require the FGP removal. The aim of this study was to evaluate the effect of the FGP removal protocol on the time for FGP removal (min), the root dentin removed (mm^3^), the angle alteration of the root canal alignment (^o^), and the root perforation occurrence using cone-beam computed tomography (CBCT) analysis. Thirty extracted maxillary molars were randomly assigned to 3 groups (n = 10): Mic-Ult, FGP removed using ultrasonic inserts under microscopic magnification; Mic-DiB, FGP removed using diamond burs under microscopic magnification, and Endo-G, FGP removed using Endo-G. CBCTs were made after access opening and after FGP removal. The volume of root dentin, the root canal alignment and root perforation were performed using InVesalius and Mimics softwares. Root canal angle alteration after FGP removal was performed using ImageJ software. The time for FGP removal was calculated in min. Data were analysed by one-way analysis of variance was followed by Tukey HSD test (α = 0.05). Endo-G resulted in significantly lower root dentin removal (*p*< .001), and significantly less time than Mic-Ult and Mic-DiB (*p*< .001). Mic-DiB had 1 and Mic-Ult had 2 root dentin perforations. FGP post removal using Mic-Ult and Mic-DiB exhibited significantly greater alteration in the root canal alignment than that using Endo-G (*p*< .001). FGP removal using Endo-G exhibited better preservation of the original canal alignment, saved root dentin structure, and also required less time compared to FGP post removal using Mic-DiB or Mic-Ult under microscopic magnification.

## Resumo

O retratamento do canal radicular e a fratura de pinos de fibra de vidro (FGP) podem exigir a remoção de FGPs. O objetivo deste estudo foi avaliar a influência das técnicas de remoção de FGP no tempo de remoção de FGP (min), no volume de dentina radicular removida (mm3), na alteração do ângulo do alinhamento do canal radicular (o) e na ocorrência de perfuração radicular usando análise de tomografia computadorizada de feixe cônico (CBCT). Trinta molares superiores extraídos foram divididos aleatoriamente em 3 grupos: Mic-Ult, FGP removido usando insertos ultrassônicos sob ampliação microscópica; Mic-DiB, FGP removido usando pontas de diamante sob ampliação microscópica, e Endo-G, FGP removido usando guia prototipado. Os CBCTs foram feitos após a abertura do acesso e após a remoção do FGP. O volume de dentina radicular, o alinhamento do canal radicular e a perfuração radicular foram realizados com os softwares InVesalius e Mimics. A alteração do ângulo do canal radicular após a remoção do FGP foi realizada usando o software ImageJ. O tempo para remoção do FGP foi calculado em min. Os dados foram analisados por análise de variância unidirecional seguida pelo teste Tukey HSD (α = 0,05). Endo-G resultou em significativamente menor remoção de dentina radicular (p< 0,001) e significativamente menor tempo para o procedimento do que Mic-Ult e Mic-DiB (p< 0,001). Mic-DiB resultou em 1 e Mic-Ult em 2 perfurações de dentina radicular. A remoção do pino FGP usando Mic-Ult e Mic-DiB exibiu alteração significativamente maior no alinhamento do canal radicular do que usando Endo-G (p<0,001). A remoção de FGP usando Endo-G resultou em maior preservação do alinhamento do canal original, preservou a estrutura da dentina radicular e exigiu menor tempo em comparação com a remoção de FGP usando Mic-DiB ou Mic-Ult sob ampliação microscópica.

## Introduction

Adhesively cemented fiberglass posts (FGP) are widely applied to restore endodontically treated teeth ^1^. However, FGP may need to be removed due to post fracture ^1^ ^,^ ^2^, or when the tooth requires endodontic retreatment ^3^ ^,^ ^4^. Mechanical removal using drills, specific devices developed by post manufacturers ^4^ ^,^ ^5^, ultrasonic inserts methods ^3^ ^,^ ^5^ ^,^ ^6^, and different types of erbium laser ^3^ ^,^ ^6^ have been indicated for FGP removal ^7^.

The FGP post removal inevitably involves loss of tooth structure, where it can be difficult to differentiate among the tooth coloured resin cement or dentin. These techniques may lead to complications such as excessive loss of dentin structure ^8^, deviations in the root canal path, root perforation ^4^ ^,^ ^9^, and heating of the periodontal ligament cells ^4^, which is particularly a concern while using ultrasonic vibrations ^6^. Operator experience can also influence the quantity of the surrounding dentin removed and the time consumed for FGP removal ^4^ ^,^ ^10^. There is a need for the FGP removal method which sacrifices less tooth structure, in order to retrieve and save the tooth ^3^.

Recently, three-dimensionally guided removal of FGP has been proposed as an alternative to conventional techniques using drills and ultrasonic inserts ^11^ ^,^ ^12^ ^,^ ^13^ ^,^ ^14^ ^,^ ^15^ ^,^ ^16^. The concept of guided endodontic was introduced in the endodontic practice to facilitate the treatment of severely obliterated teeth ^17^ ^,^ ^18^ ^,^ ^19^. This technique uses a 3-dimensional (3-D) imaging data obtained by cone-beam computed tomography (CBCT), surface scanning of teeth or models, and virtual planning to obtain a prototyped guide aiming to locate root canals and perform an optimized access cavity preparation ^18^ ^,^ ^20^. According to the current literature, this method shows high accuracy ^15^ ^,^ ^16^ ^,^ ^21^ ^,^ ^22^, both *in vitro* ^20^ ^,^ ^21^ ^,^ ^22^ and in *ex vivo* ^23^. Clinical reports have confirmed the usefulness of guided endodontics for the canal partially or entirely obliterated ^11^ ^,^ ^24^.

The use of 3-D printed guides to remove the FGP, providing a new protocol to address the challenges of FGP removal has been demonstrated in laboratory studies ^13^ ^,^ ^14^ ^,^ ^15^ ^,^ ^16^ and case reports ^24^ ^,^ ^25^. Although these guides have been credited with being able to orient the access cavity for FGP removal more safely, contributing to the preservation of root structure ^17^ ^,^ ^18^, there is a scarcity of studies that evaluate this method in comparison with other commonly used methods. Considering that guided FGP removal requires extra patient appointment, consume time needed to fabricate the appliance, add costs for final endodontic procedure and also is not yet easily available to all professionals requiring special training ^12^, the effectiveness to prevent the loss of intact root dentin and the alteration of the canal alignment resulting from FGP removal using this method should be more investigated. The analysis of the time consumed, and the accuracy of the 3-D printed guides compared with ultrasonic inserts and diamond burs ^21^ ^,^ ^22^ ^,^ ^26^ ^,^ ^27^, need to be investigated to better orientate the clinicians for viability of the additional cost involved with protocols to remove the FGP.

The dentin loss, and canal transportation caused by FGP removal can be measured using the reconstructed 3D models created by micro-CT and CBCT images ^5^ ^,^ ^8^ ^,^ ^23^. The micro-CT is very effective when calculating the small volume within the tooth ^3^. However, the CBCT method is evaluable in the clinical routine. Therefore, the aim of this in vitro study was to evaluate the time required for FGP removal, dentin loss, and canal transportation resulting from FGP removal using endodontic guide, ultrasonic inserts, or diamond burs. The CBCT was used in combination with 3D reconstruction software analysis for this evaluation. The null hypothesis was that the use of endodontic guide would not reduce the time required for FGP removal and would not influence the dentin removal and the root canal alignment among the tested methods.

## Materials and Methods

### Specimen preparation

Thirty freshly extracted maxillary first permanent human molars with fully formed apices were used in accordance with the guidelines suggested by the local ethics committee (protocol 85533718.1.0000.5152). The teeth were collected, maintained in distilled water with Timol during the storage time, for no more than 30 days. Sample sizes required to detect a difference of 30% considering 3 groups, given a significance level of 5% and a power of 80% were calculated resulting in n = 10.

**Figure 1.**
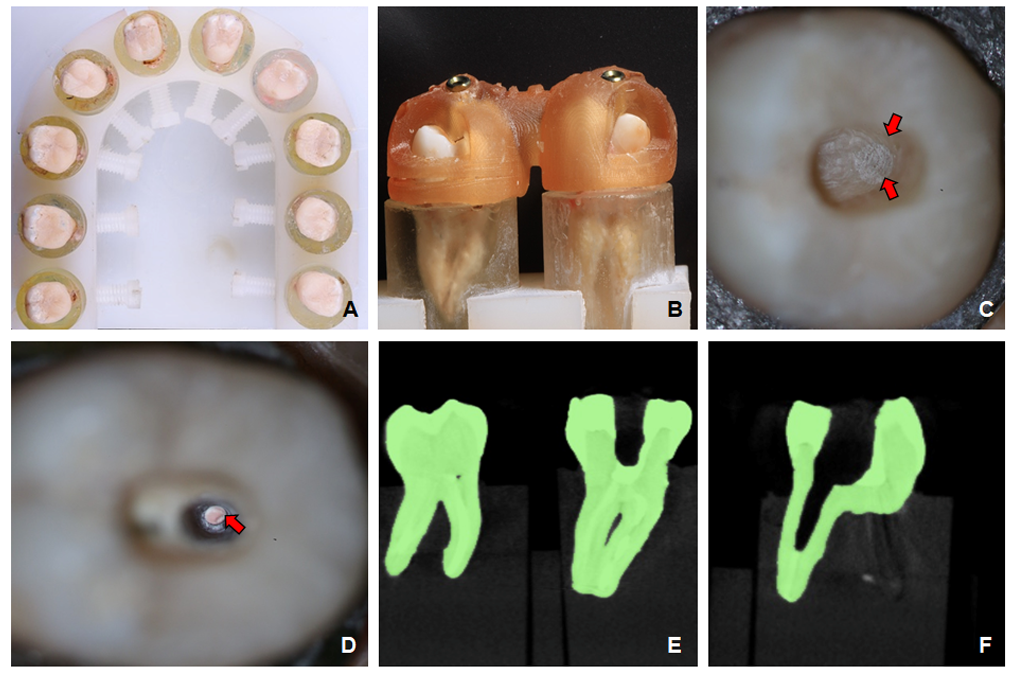
Specimen preparation. A, Polyacetal device with 10 samples fixed for endodontic access and FGP removal; B, prototyped printed guide installed and fixed on prepared specimens; C, root canal access finalized with visualization of FGP (red arrow); D, FGP removal finalized with visualization of gutta-percha (red arrow); E, three-dimensionally reconstructed images of specimens after root canal access; F, three-dimensionally reconstructed images of specimens after FGP removal.

The lingual cusps and the coronal portion of the teeth were removed, followed by removal of the pulp roof, simulating the structure of compromised teeth requiring endodontic treatment. The teeth were embedded in polystyrene resin (Crystal; Redelease) and mounted in a polyacetal device designed for this study, which allowed stabilization and standardization of the positions during all procedures (Figure 1).

### Endodontic treatment

Endodontic treatment was performed using a standard preparation for all teeth using nickel-titanium rotary system (Logic Easy; Easy Equipamentos). Apical patency was maintained with a #10 hand file. Root canal irrigation was performed using 2.5% NaOCl (Sodium Hypochlorite; Asfer) with a syringe and endodontic needle (Navitip; Ultradent). The root canal cleaning was performed with ethylenediaminetetraacetic acid 17% (EDTA; Maquira) activated using cleaning files (Easy Clean; Dentsply Sirona). The final irrigation with distilled water, followed by drying the canals with paper cones #40 (Absorbent Paper Points; Dentsply Sirona). Obturation was made utilizing a unique gutta-percha accessory cone (M size Guttapercha Accesory Cone; Dentsply Sirona) associated with endodontic resin cement (AH Plus; Dentsply Sirona). The canal was irrigated with ethylenediaminetetraacetic acid 17% (EDTA; Maquira) activated with a flexible instrument (EasyClean; Easy Equipment), and finally irrigated with distilled water then dried with paper cones (Absorbent Paper Points; Dentsply Sirona). The canals were filled with gutta-percha and a resin-based sealer (AH Plus; Dentsply).

### FGP cementation

The palatal canal was standardly prepared using a specific drill of FGP system (#2 drill, Whitepost System; FGM) up to 8 mm of the root length considering the pulp floor as a reference, leaving at least 5mm of endodontic filling material. A dual conic/cylindrical FGP systems (#2, Whitepost System; FGM), that has 1.8 mm in diameter at cervical portion and 0.5 mm at apical limit. The FGP was cleaned with 70% alcohol (Alcohol; Alkofarma). The silane (Prosil; FGM) was actively applied for one minute and dried using air spray application. The canal was filled with self-adhesive resin cement (Rely U200; 3M Oral Care) with an endo tip ^28^. The FGP was inserted into the root canal with digital pressure, the resin cement excess was removed, and after waiting for 5 min was light-cured for 40 s ^29^. The FGP was cut at the dentin-enamel limit using diamond bur (#1014, KG Sorensen, Barueri, SP, Brazil). The adhesive system (Ambar Universal APS; FGM) was actively applied into the cavity preparation surface, followed by gentle air spray. The adhesive system was photo-activated for 20 s using a light curing unit (VALO Grand; Ultradent) with irradiance of 939 mW/cm^2^ measured using an integrating sphere (Labsphere) connected to a fiber-optic spectroradiometer (USB 4000; Ocean Insight). After the adhesive system application and photo-activation, the bulk-fill resin composite (OPUS Bulk fill APS; FGM) was inserted and condensed into the root canal in two increments of 5.0 mm. The bulk-fill RBC increments were photo-cured for 40 s. The initial CBCT images were obtained (3D XP 68; PreXion) at 110 kV and 6.19mA using a field of view of 5 cm x 5 cm, using Endo Mode (Ultra High Definition) with 20 seconds, gantry rotation period 120 ms; 0.1-mm voxel size; resolution 512×512 pixels.

### FGP removal and time consume calculation

Endodontic access opening of all teeth was performed using a round diamond bur (#1014; KG Sorensen) and Endo-Z bur (Prima Dental; Gloucester) to gain access to the FGP at the entrance of the root canal. The CBCT images were obtained after access opening using the same protocol. The teeth were randomly allocated to 3 groups according to the FGP removal technique, using a randomization computer-generated system (https://www.sealedenvelope.com/).

*Mic-Ult group,* the FGP was removed using ultrasonic inserts (E3D and E2D; Helse Ultrasonic) coupled to an ultrasonic device (P5, XS; Newtron Satelec) under clinical microscopic magnification (OPMI Pico; Zeiss) with 12.5x magnification. One ultrasonic insert was used for each 5 teeth.

*Mic-DiB group,* FGP was removed using a round diamond bur with 0.9 mm in diameter (1011 HL; KG Sorensen) coupled to a high-speed handpiece (Friction Grip, Dabi-Atlante) with continuous irrigation under clinical microscopic magnification (OPMI Pico; Zeiss) with 12.5x magnification. One round diamond bur was used for each 5 teeth.

*Endo-G group,* the FGP was removed using a prototyped endodontic guide with the drill diameter adjusted according to the length and diameter of the endodontic post (Ref 103.395, Neodent) at 950rpm and 5Ncm torque. One drill was used for each 5 teeth. Initially, the teeth were scanned to generate a stereolithographic file, which was uploaded to a guided implant software (CoDiagnostiX; Dental Wings). Access was planned for all samples with a 1.3-mm-diameter drill (Ref 103.395, Neodent) from the beginning to the end of the post. Three-dimensional printing of the guide was performed (Moonray DLP 3D Printer, Sprintray) in translucent resin (Surgical guide; Sprintray Inc). The guide was tested in the model to assess any alteration that could change the accuracy of the guided access.

FGP post removal was confirmed when gutta-percha was detected. The time required for FGP removal was recorded for all specimens. The CBCT images were obtained again after FGP removal.

### Root dentin removal and canal alignment analysis

The tooth structure wear captured by CBCT was evaluated using the InVesalius software (InVesalius; Renato Archer Information Technology Centre) installed on an Air operational system Sierra 10.12.6 (MacBook; Apple Inc). The threshold was established by gradually increasing the minimum density value until the support was completely excluded from the image. The initial and final volumes of enamel and dentin of each tooth were calculated in cubic millimetres. The volume of tissue removed during FGP removal was calculated using the following formula: Vr = Vt - Vf (Vt is the volume of the tooth and Vf is the volume of the tooth after FGP post removal).

The scanned CBCT images were used for the reconstruction of a 3D model of the different hard tissues identified using an interactive medical imaging software (Mimics 16.0; Materialize). Segmentation of tooth structures and restorative materials was accomplished based on image density thresholding. The manual threshold for each structure was defined choosing the range of the pixel value that highlighted each structure automatically. The segmentation was confirmed using the Mimics library of the mask for enamel and dentin. The reconstructed virtual 3D models obtained before and after FGP removal were used to define the root alignment. The initial root canal alignment was traced from the center of FGP alignment creating a red full line. The center of the apical preparation and the coronal center point of the FGP were used to trace the root canal alignment after FGP post removal creating a dotted green line. The angle generated between the alignment of these two lines was calculated using a public domain software (ImageJ, National Institutes of Health, Bethesda, MD, USA). The presence of root canal perforation was evaluated by three experienced professionals blinded for FGP removal technique.

### Statistical analysis

The data regarding the required time (minutes) for FGP removal, the volume of removed dentin (mm^3^), and the angle alteration alignment (^o^), were tested for normal distribution (Shapiro-Wilk test) and equality of variances (Levene test), followed by parametric statistical tests. One-way analysis of variance was followed by Tukey’s test. Statistical significance was set at α = 0.05. All statistical analyses were performed using Sigma Plot (version 13.1; Systat Software Inc).

## Results

The time required for FGP removal for all groups are shown in Table 1. FGP removal using microscope and ultrasonic inserts required significantly more time than that using microscope and diamond burs (*p*< .001). The use of Endo-G required significantly less time for FGP removal than the other 2 techniques (*p*< .001).

**Table 1:** Time (minutes) required, the volume of root dentin removed (mm^3^), and the angle root canal alignment alteration (^o^) and standard deviations after FGP removal using 3 protocols.

FGP removal protocols	Time required for FGP removal (min)	Volume of root dentin removed (mm^3^)	Root canal alignment alteration (^o^)
Endo-G	7.6 ± 3.1 A	5.3 ± 2.1 A	0.5 ± 0.2 A
Mic-Ult	33.7 ± 13.3 C	13.2 ± 3.0 B	2.3 ± 0.9 B
Mic-DiB	12.9 ± 5.3 B	21.8 ± 6.7 C	2.7 ± 1.2 B

Different letters indicate significant differences among tested protocols according to Tukey’s test with statistical significance set at *P* <0.05.

The volume of resin composite removed during FGP removal for all groups are shown in Table 1. FGP removal using microscope/diamond burs resulted in significantly more root dentin removal than that using microscope/ultrasonic inserts (*p*< .001). The use of Endo-G resulted in a significantly smaller volume of root dentin removal than the other 2 techniques (*p*< .001).

**Figure 2:**
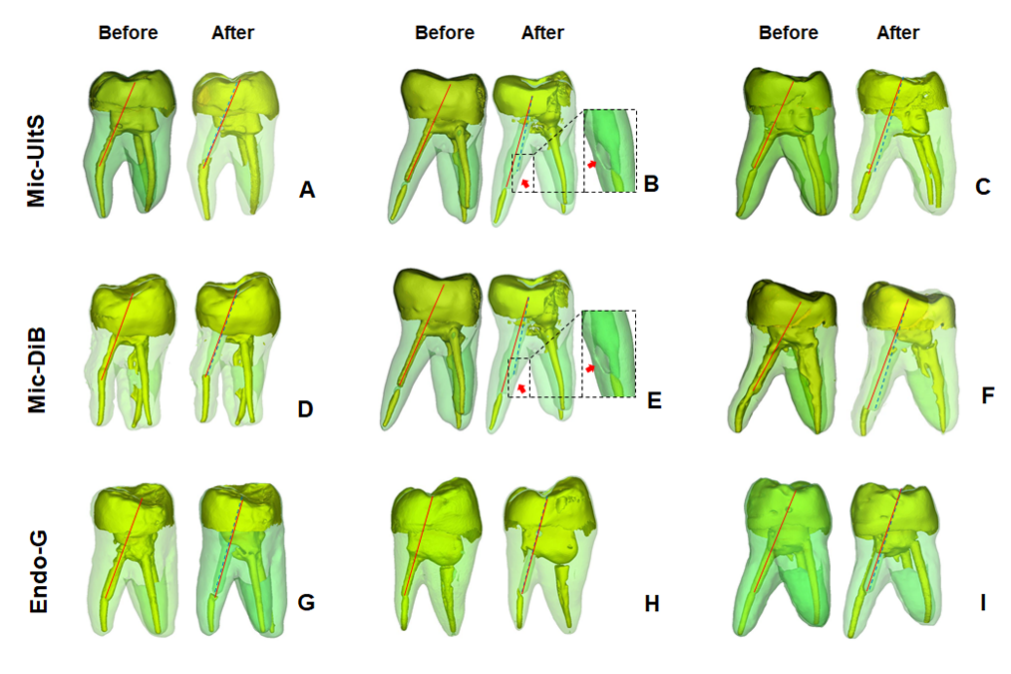
Representative three-dimensional images of specimens before and after root canal access and FGP removal using all protocols. A, Mic-Ult group shown root removal closer to pulp chamber; B. Mic-Ult root group shown root alignment alteration and root dentin perforation (red arrow); C, Mic-Ult root group shown root removal closer to pulp chamber and small root alignment alteration; D, Mic-DiB group shown great amount of dentin removal along to the root canal preparation; E. Mic-DiB group shown great amount of dentin removal along entire root canal preparation and also alignment alteration resulting in root perforation (red arrow). F. The Mic-DiB group showed a great amount of dentin removal along the entire root canal preparation and also alignment alteration; G H and I. Endo-G group showed consistently less root removal along the entire root canal. Red full line was used to show the initial root canal alignment and dotted green line was used to show the final root canal alignment after FGP removal.

The angle alteration (^o^) of the root canal after FGP removal for all groups are shown in Table 1. FGP post removal using Mic-Ult and Mic-DiB exhibited significantly greater angle alteration in the root canal alignment than that using Endo-G (*p*< .001). The representative 3D reconstruction of specimens, the frequencies of root canal transportation, root canal perforation, and from all groups before and after FGP removal are shown in Figure 2. FGP removal using Mic-Ult resulted in 2 root perforations and Mic-DiB resulted in 1 root perforation. Endo-G resulted in no root perforation.

## Discussion

The null hypothesis was rejected, as guided access outperformed the other two techniques in the investigated parameters. This method was compared with ultrasonic devices and burs, which are generally used for FGP removal ^4^ ^,^ ^7^. Recent clinical reports have demonstrated that FGP removal using an endodontic guide is safer, maintains the axis of the drill in the root canal axis, and contributes to the preservation of root structure ^11^ ^,^ ^12^. However, there are no clinical studies supporting such claims, and to the best of our knowledge, there are no previous laboratory studies comparing the superiority of guided FGP removal compared to other traditionally used methods.

The removal of FGP using conventional diamond burs is usually carried out through a hole in the centre of the post and large-diameter drills are used for additional milling ^5^. This process is prone to accidents, since it is difficult to control high-speed burs within the small confines of the root canals and the burs tend to slip off surfaces ^4^. In the present study, a high volume of surrounding dentin was removed in the Mic-DiB group. This protocol also altered the root canal alignment and resulted in root canal perforation in one of the specimens. Additionally, the ultrasonic insert protocol required significantly more time than the other techniques and resulted in root dentin perforation in two specimens. In fact, post removal with ultrasonic tips may require more time than that with the drill method, which is partly confirmed by the current literature ^5^ ^,^ ^6^. Moreover, they require clinical experience, familiarity with handling ultrasonic devices ^10^, and the use of magnification ^8^.

Even for an experienced endodontist using an operating microscope, conventional access with diamond burs or ultrasonic inserts resulted in greater dentin wear, greater alteration of the root canal alignment, and higher incidence of root dentin perforation during post removal when compared with guided removal. Previous study evaluating the use of endodontic guides for access to simulated calcified canals demonstrated lower dentin loss, lower working time, and higher accuracy of canal localization than the conventional technique, regardless of previous professional experience ^22^. Although the present study did not compare the performance of FGP removal methods according to the operator’s skills, it is evident that the use of virtually guided planning for FGP removal significantly decreases the chances of iatrogenic occurrences. It is important to address that all procedures were performed by a single professional, with more than 10 years of experience in endodontics, which can be considered a factor able to influence the results, as previously demonstrated in a study evaluating removal of FGP by ultrasound and drills ^10^. Probably, a less-experienced operator would remove more dentin during FGP removal than a skilled operator, due to less sensitivity, despite the used technique, resulting in a worse scenario. In such a scenario, guided FGP removal could be even more beneficial than this study has shown.

Guided endodontics has emerged as an alternative technique in complicated situations of FGP removal, resulting in a faster and more secure procedure than the conventional methods ^11^ ^,^ ^12^ ^,^ ^13^ ^,^ ^14^ ^,^ ^15^ ^,^ ^16^. However, it is important to emphasize that this method can also result in deviation of the root canal axis, as observed in one of the samples. Thus, it is extremely important to obtain radiographic images during post removal to avoid catastrophic consequences. Another point of interest in the present study was to determine the role of access cavity in total removal. CBCT images obtained after designing the access cavity revealed no significant differences among the groups at this stage. Although pre-clinical studies have used CBCT images for the analysis of canal deviation and changes in dentin volume due to post removal ^5^ ^,^ ^8^, CBCT was utilized because it is the imaging examination used for planning the treatment.

Despite the encouraging findings from studies employing guided endodontics ^17^, it's essential to address certain methodological limitations. The study involved human molars, and despite the careful selection of teeth, variations in root canal anatomy are anticipated, potentially influencing the results. This issue could be mitigated by using standardized resin printed teeth, which would also eliminate variations in enamel-to-dentin composition and ratio, facilitating the calculation of tooth substance loss ^19^. However, the installation of FGP in printed resin teeth would not yield the same level of adhesion as seen with human dentin, potentially leading to easier FGP removal using drills or ultrasonic inserts. Another important issue that should be considered is the limited accuracy of the CBCT used to measure the submicrometric dimensions involved during the clinical procedure of FGP removal. Voxel size of the CBCT can influence the characteristics of the final image. The voxel will then reflect the average density, causing limitation to detect the true value of either objects ^30^. CBCT imaging can be used for endodontic measurement, however the smallest voxel size and highest resolution yielded more accurate results. ^31^ The use of microCT methodology can contribute with more precise analysis of the parameters measured in this study ^32^.

This study has some limitations, this study tested only one type of FGP, the translucency and the diameter can influence the parameters measured. The absence of a model that mimics insertion of the endodontic guide in the upper molar region was another limitation of the present study. Since the samples were not inserted in jaw models, post removal might have been facilitated, reducing the total time required for the procedures. However, this might not have led to inaccuracies, as all teeth were prepared in a similar manner. Future studies must analyse the real impact of the amount of the dentin loss during the FGP removal on the biomechanical performance of the endodontic treated molar teeth.

Another point that requires attention is the substantial amount of residual debris on the root canal walls observed in three specimens after utilizing guided endodontics for post removal. These residues were also observed in the drill group and the ultrasonic group, as previously demonstrated by other studies ^26^ ^,^ ^27^. Most of the FGP removal techniques leave undesirable residues of cement, gutta-percha, or FGP on the dentin walls ^5^ ^,^ ^9^ ^,^ ^26^ ^,^ ^27^. Thus, guided post removal requires some improvements such as the development of special drills for differential cutting of FGPs and intermediate restoration, which allows better irrigation and avoids temperature increase ^12^, and specific design for the posterior teeth due to limited accessibility in the posterior area ^20^ ^,^ ^21^.

## Conclusion

Within the limitations of this in vitro study design, it is possible to conclude that the guided endodontic access consumed significantly lower treatment time, reduced the dentin removal, and produced less root canal alignment alteration and significantly lower incidence of root perforation compared microscope/ultrasonic inserts and microscope/diamond burs for FGP removal (*p* <.001).
